# Microemulsions Enhance the In Vitro Antioxidant Activity of Oleanolic Acid in RAW 264.7 Cells

**DOI:** 10.3390/pharmaceutics14102232

**Published:** 2022-10-19

**Authors:** Chiara De Stefani, Marzia Vasarri, Maria Cristina Salvatici, Lucia Grifoni, Jose Carlos Quintela, Anna Rita Bilia, Donatella Degl’Innocenti, Maria Camilla Bergonzi

**Affiliations:** 1 Department of Chemistry, University of Florence, Via Ugo Schiff 6, Sesto Fiorentino, 50019 Florence, Italy; 2 Department of Experimental and Clinical Biomedical Sciences “Mario Serio”, Viale Morgagni 50, 50134 Florence, Italy; 3 Institute of Chemistry of Organometallic Compounds (ICCOM)-Electron Microscopy Centre (Ce.M.E.), National Research Council (CNR), Via Madonna del Piano 10, 50019 Sesto Fiorentino, Italy; 4 NATAC BIOTECH, Electronica 7, Alcorcón, 28923 Madrid, Spain

**Keywords:** oleanolic acid, microemulsions, PAMPA, LPS, RAW 264.7 murine macrophages

## Abstract

Oleanolic acid (OA) is the main triterpenic acid of olive leaves known for numerous pharmacological properties, including antioxidant activity. However, it is poorly soluble in water and consequently with low bioavailability, which limits its pharmacological application. Microemulsions (MEs) are dispersed systems consisting of two immiscible phases that promote rapid solubilization and absorption in the gastrointestinal tract. To improve both solubility and intestinal permeability of this molecule, OA has been formulated in two different microemulsions (ME-1 and ME-2). A solubility screening was carried out to select the ME components, and pseudoternary phase diagrams were constructed to evaluate the region of existence and select the appropriate amount of the constituents. ME-1 was prepared using Capmul PG-8/NF as the oily phase, and Transcutol and Tween 20 (7:3) as surfactants, while ME-2 contained Nigella oil and Isopropil myristate as the oily phase, and Transcutol HP and Cremophor EL (2:1) as surfactants. The OA solubility was increased by 1000-fold and 3000-fold in ME-1-OA and ME-2-OA, respectively. The MEs’ droplet size and the PdI were evaluated, and the stability was assessed for 8 weeks by monitoring chemical and physical parameters. The parallel artificial membrane permeability assay (PAMPA) also demonstrated an enhanced intestinal permeability of both OA formulations compared with free OA. The potential ability of both MEs to enhance the bioactivity of OA against LPS-induced oxidative stress in RAW 264.7 murine macrophages was also investigated. Overall, this study suggests that both MEs promote a bio-enhancement of the protective action of OA against the LPS-induced pro-oxidant stress in macrophages. Overall, this study suggests that MEs could be an interesting formulation to improve OA oral bioavailability with potential clinical applications.

## 1. Introduction

Chronic and noncommunicable diseases generally have a multifactorial etiology, and thus are often caused by a concomitance of multiple modifiable risk factors. However, oxidative stress and inflammation are common denominators in the vast majority of these diseases [1,2]. In the inflammatory response, the affected regions become filled with leukocytes and mast cells, causing a “respiratory burst” or increasing oxygen uptake, which lead to the production and release of reactive oxygen species (ROS) in the damaged area [3]. ROS are continuously formed as natural byproducts of physiological cellular activity and participate in cell signaling [4]. Healthy cells balance the formation and elimination of ROS by maintaining their homeostasis. However, when ROS levels exceed a critical threshold, homeostasis is disturbed, with detrimental effects on cellular structures and functions and subsequent oxidative stress. As such, the disturbance of cellular redox balance is a risk factor for the development of various diseases [5]. This is why countering excessive ROS formation may contribute to the reducing burden of chronic diseases. In this context, antioxidant strategies offer some important opportunities to prevent or block this pathophysiological chronic phenomenon.

Plant-derived phytochemicals have been shown to exert a protective effect against the development of these diseases due to their antioxidant properties [6]. So far, about 25,000 phytochemicals have been discovered, and still a large percentage remains unknown. These phytochemicals include polyphenols, tannins, flavones, carotenoids, phytosterols, steroids, saponins, alkaloids, and triterpenoids [7].

However, many drugs and herbal extracts, despite their bioactivities in experimental in vitro models, show less or negligible efficacy in vivo due to their poor solubility or improper molecular size, resulting in poor absorption and thus poor bioavailability. In the case of herbal extracts, there is a great possibility that many compounds are partially or entirely destroyed in the highly acidic pH of the stomach. Other components may be metabolized partially or entirely by the liver before reaching the bloodstream. As a result, the actual amount of the drug may not reach the blood entirely. Nowadays, with the advancement of technology, novel drug delivery systems open the door towards the development of enhanced bioavailability of herbal drugs. Nanotechnology is a rapidly evolving field that also provides great benefits in phytomedicine [8,9,10]. The incorporation of nano-based formulations has brought advantages in herbal formulations, including improved bioavailability and solubility, protection from physicochemical degradation, increased therapeutic activities, enhanced stability, and sustained delivery. Therefore, nano-phytomedicine offers great promise for solving problems associated with herbal medicine [11]. Drug delivery systems are used to improve the solubility and increase the bioavailability of poorly water-soluble drugs [12,13]. In recent years, numerous techniques have been developed to improve the oral bioavailability of poorly water-soluble drugs, including mesoporous silica, microemulsions [14], self-nano-emulsifying drug delivery systems [15], liquid crystalline nanoparticles [16], nanoengineered mucopermeating drug delivery systems [17], and lipid nanoparticles [18]. The lipid-based drug delivery system is another very promising approach to improve the solubility, absorption and consequently bioavailability of drugs with poor aqueous solubility. Lipid-based formulations for oral administration have a wide diversity, ranging from simple oil solutions to complex mixtures of surfactants, co-surfactants or co-solubilizers and oil. Self-nanoemulsifying drug delivery systems (SNEDDS) are an example of the latter type [19]. These are anhydrous preconcentrates of nanoemulsions that, when administrated into aqueous phase and followed by mild agitation due to gastric motility, form oil-in-water nanoemulsions. Self-nanoemulsifying drug delivery systems (SNEDDS) have shown exponential increase from the formulator’s perspective. SNEDDS have demonstrated wide applicability in terms of the controlled and targeted delivery of various types of drugs [15].

Oleanolic acid (OA) is a naturally occurring pentacyclic triterpenoid isolated from several food and medicinal plants [20]. *Olea europaea* L., the plant species from which the compound takes its name, is still the primary source of OA in the family Oleaceae. Many studies attribute to OA numerous pharmacological properties that have made it of great interest for its therapeutic potential in a variety of chronic human pathologies [21,22]. In numerous in vitro and in vivo animal model studies, OA has shown antioxidant, anti-inflammatory, neuroprotective, hepatoprotective, anti-hyperlipidemic, anti-osteoporotic, anticancer and antibacterial properties [21,22,23]. Folk medicine uses OA in many ways [24]; in addition, OA is marketed as a remedy for various disorders under different formulations [25]. The ability of OA to act through complex, multifactorial biomechanisms by regulating molecular interactions and multiple signaling pathways justifies the numerous biological activities of this pentacyclic triterpenoid [21].

Despite its innumerable biological effects, OA has poor aqueous solubility (1.75 μg/L) with low bioavailability. OA oral bioavailability is only 0.7% for oral doses of 25 and 50 mg/kg in rats; this might be due to its poor solubility and dissolution rate [26]. Therefore, its use in the pharmaceutical field is rather limited [23].

Microemulsions (MEs) are dispersed systems consisting of two immiscible phases which spontaneously form with no energy under aqueous titration method. They usually have a mean diameter from 10 to 100 nm [27]. As they are liquid formulations, the oral route is the ideal route of administration, therefore favoring rapid solubilization and absorption in the gastrointestinal tract. Compared with other lipid carriers, MEs offer the advantages of high interfacial area, transparency, low viscosity, long-term stability, potential for transport of both hydrophilic and hydrophobic drugs, higher drug stability, enhanced transmucosal and transdermal drug delivery, nanoparticle fabrication, and thus better bioavailability. Furthermore, nanoemulsions have remarkable wetting, spreading and penetration abilities and can be scaled up due to ease of manufacture. Some factors limiting their use are poor palatability, hydrolysis of drugs due to lipid content, higher water content and long-term storage [19]. SNEDDS, encapsulated as single, water-free dosage forms, offer improved physical and chemical stability, palatability, and patient compliance. They are able to improve permeability and have high drug-loading capacity compared with lipid solutions due to the high concentration of surfactants and co-surfactants. However, stability issues, such as drug precipitation during storage and incompatibility of ingredients in shell, decrease their applicability. Lipid nanoparticles are stable formulations [28]. This aspect is of paramount importance compared to colloidal drug carriers. They show excellent reproducibility with production by high-pressure homogenization suitable for scaling up. They have a wide range of potential applications, such as intravenous, cutaneous, oral and topical. However, solid lipid nanoparticles have limited drug-loading capacity, drug loss during storage, and relatively high water content. Nanostructured lipid carriers have been developed for better drug accommodation to increase drug loading and prevent drug expulsion.

In this work, MEs were selected as nanosized drug delivery systems to increase OA aqueous solubility and therefore its bioavailability. Two different MEs were fully characterized, assessing chemical and physical parameters and release characteristics, and their stability was evaluated in simulated gastro-intestinal fluids and for two months’ storage at 4 °C and 25 °C. Furthermore, the ability of the two MEs to improve intestinal permeability was evaluated using PAMPA (parallel artificial membrane permeability assay). The potential ability of both MEs to enhance the OA activity against lipopolysaccharide (LPS)-induced oxidative stress in RAW 264.7 murine macrophages was also investigated. LPS is a widely used stimulus to activate macrophages; through the production of pro-inflammatory mediators, LPS causes oxidative stress [29,30]. Upon LPS stimulation, macrophages reprogram their metabolism by driving the generation of ROS, which are thought to be involved in the mechanism of LPS-induced cellular toxicity [31].

## 2. Materials and Methods

### 2.1. Chemicals and Reagentds

Oleanolic acid (OA) was provided by Natac (Madrid, Spain). Acetonitrile HPLC grade, Cremophor EL, DMEM, Ethanol, Etyl oleate, Fetal Bovine Serum. Formic acid, Methanol HPLC grade, Oleic acid, PEG400, Soybean oil, 1,7-octadiene, Tween^®^ 20 and Tween^®^ 80 were purchased from Merck KgaA (Darmstadt, DA, Germany). Capmul PG-8 NF, Capmul PG-12 NF, Capryol 90, Labrafac lipophile, Labrasol, Labrasol ALF, Lauroglycol 90, Peceol, Plurol oleique CC 497, and Transcutol HP were supplied by Gattefossè sas (Saint-Priest, France). Captex 300 and Captex 355 were purchased from Abitec. Phosphotungstic acid was purchased from Electron Microscopy Science (Hatfield, MA, USA). The water used was from the Milli-Qplus system from Millipore (Milford, CT, USA). Sodium dodecyl sulfate (SDS) was from Merck (Rome, Italy). The dialysis kit was from Spectrum Laboratories, Inc. (Breda, The Netherlands). The PAMPA filter plate (pore size 0.45 μm) was purchased from Millipore Corporation (Tullagreen, Carrigtwohill, County Cork, Ireland). Nigella oil was supplied by Biokyma srl (Anghiari, AR, Italy). Isopropyl myristate was purchased by Galeno srl (Prato, Italy). Merck KgaA (Darmstadt, DA, Germany) provided the necessary components for cell culture and cell-based in vitro experiments: DMEM (Dulbecco’s Modified Eagle Medium) culture medium, penicillin and streptomycin, L-glutamine, FBS (Fetal Bovine Serum), trypsin-EDTA solution, Phosphate Buffered Saline (PBS), 1-(4,5-dimethylthiazol-2-yl)-3,5-diphenyl formazan (MTT), 2′,7′-Dichlorofluorescin diacetate (DCFDA) fluorescent probe, lipopolysaccharide (LPS, from *E. coli* O111:B4). Sterile disposable plastic was purchased from Sarstedt (Verona, VR, Italy).

### 2.2. Cromatography Conditions and Instruments

The HPLC system consisted of a 1200 High Performance Liquid Chromatograph (HPLC) equipped with a Diode Array Detector (DAD) from Agilent Technologies Italia Spa (Rome, Italy). The analytical column was a Luna Omega Polar C18 (150 × 4.6 mm, 3 μm) (Agilent Technology, Santa Clara, CA, USA). The compounds were detected at 210 nm with an eluent flow rate of 0.5 mL/min, with (A) acetonitrile and (B) water pH 3.2 (by formic acid) as mobile phases, and with an isocratic analytical method consisting of 80% A and 20% B. The calibration curve, with a coefficient of determination R^2^ of 0.9999, was prepared using a standard solution of OA in methanol (2.0 mg/mL) and successive dilutions of 2-, 5-, 10-, 20-, 50-, 100-, 200- and 250-fold.

### 2.3. Preparation of Microemulsions

#### 2.3.1. Solubility Study

The solubility of OA in different vehicles was defined by adding an excess of OA to 2 mL of each of the tested solvents/tensides: Water, Capmul PG-8/NF, Capmul PG-12/NF, Capryol 90, Captex 300, Captex 355, Cremophor EL, Etyl Oleate, Isoprpyl myristate, Labrafac lipophile, Labrafilm 1944, Labrafilm 2125, Labrasol, Labrasol ALF, Lauroglycol 90, Nigella Oil, PEG400, Soybean Oil, Sunflower Oil, Transcutol, Triacetine, Tween 20 and Tween 80. Each mixture of solvent and OA was stirred for 24 h at 25 °C, then it was centrifuged at 14,000× *g* for 10 min. After removing the precipitate, the supernatant was diluted with MeOH and analyzed by HPLC-DAD to determine the concentration of the OA. The analyses were performed in triplicate.

#### 2.3.2. Pseudoternary Phase Diagram

The pseudoternary phase diagram was constructed in accordance with the water titration method, to define the area of existence of the MEs. The selected surfactants were mixed at various ratios (Smix), then the pseudoternary phase diagrams were built using different weight oil-phase/Smix ratios: 10:90, 20:80, 30:70, 40:60, 50:50, 60:40, 70:30, 80:20, and 90:10. Each of the mixtures obtained were tested by adding water dropwise to each blend under magnetic stirring at room temperature. During water addition, the change in sample appearance was monitored to determine if transparent ME, emulsion, or gel-like structures were present.

#### 2.3.3. Preparation of MEs

The two MEs were prepared following the water titration method [32,33], adding water dropwise to each oily phase/Smix blend. For the first ME (ME-1), the lipophilic phase was obtained by mixing, under constant magnetic stirring at 50 ± 2 °C, Transcutol HP and Tween 20 at a 7:3 ratio with Capmul PG-8/NF, obtaining a final 9:1 Smix/Oil ratio. The resulting blend was maintained under stirring at room temperature for 15 min. For the second ME (ME-2), the lipophilic phase was obtained by blending, under stirring at 70 ± 2 °C, Transcutol HP and Cremophor EL at a 2:1 ratio with Isopropil myristate and Nigella sativa oil, respectively, at a 2:1 weight ratio. The resultant weight ratio Smix/Oil was 12.5:1. OA-loaded MEs were obtained by adding OA to the Smix/Oil blend before titration. The obtained blend was titrated with water at 35 ± 2 °C. The resulting ME was maintained under stirring at room temperature for 15 min.

### 2.4. Characterization of Microemulsions

#### 2.4.1. Particle Size and ζ-Potential Measurements

Droplet sizes of the developed MEs were evaluated by dynamic light scattering (DLS), using Ζetasizer Pro Red Label (Malvern Instruments, Malvern, UK) at 25 °C. The hydrodynamic diameter of the particles and the particle size distribution (polydispersity index, PdI) were obtained using the ZS Xplorer software provided by Malvern. Scattering was measured in a 4 mL borosilicate cell at a 90° angle, diluting the samples in distilled water. ζ-potential was measured using the same instrument; for all samples, an average of three measurements was taken. The temperature was maintained constant at 25 °C by a temperature controller.

#### 2.4.2. Morphological Characterization

The samples were analyzed using the Scanning Electron Microscope Gaia 3 (Tescan s.r.o, Brno, Czech Republic) FIB-SEM (focused ion beam-scanning electron microscope). The electron beam used for TEM (transmission electron microscope) imaging had a voltage of 15 kV, operating in high-vacuum mode and with a bright-field TEM detector. The MEs were diluted 10-fold with deionized water, and 10 μL was applied to a 150-mesh carbon film-covered copper grid. To obtain a thin film, excess sample was eliminated from the grid with a filter paper. After that, 5 μL of phosphotungstic acid solution (1% *w/v* in water) was dropped onto the grid as a staining medium and the excess solution was removed with a filter paper. Samples were dried for 3 min, after which they were examined with the electron microscope and photographed at an accelerating voltage of 20 kV.

#### 2.4.3. Chemical and Physical Stability during Storage

To estimate storage stability, the OA-MEs were stored at 4 °C and 25 °C for two months. Chemical and physical stabilities were checked periodically by changes in terms of particle size, homogeneity, ζ-potential, and OA concentration by DLS and HPLC-DAD analyses.

#### 2.4.4. In Vitro Release Studies

The release studies of OA from MEs in comparison to OA solution (SDS 0.5% p/V in H_2_O) were carried out with the dialysis bag method (regenerated cellulose dialysis membranes, Spectrum Laboratories, Inc., Breda, The Netherlands, MWCO 12–14 kD). Two mL of the OA-MEs or the solution were placed into a 12–14 kDa dialysis membrane and immersed into 200 mL of the release medium at 37 °C under magnetic stirring, using EtOH:PBS (30:70) as the release medium. In vitro release study was also carried out in the simulated gastric fluid (SGF) medium at pH 1.2 for 2 h followed by simulated intestinal fluid (SIF) at pH 6.8 for 6 h. The composition of the gastric fluid was 2 g of NaCl and 7 mL of HCl per liter of deionized water. The intestinal fluid was composed of 6.805 g of KH_2_PO_4_ and 0.896 g of NaOH per liter of deionized water [32,34]. At predetermined time intervals, 1 mL of each release medium was withdrawn and replaced with an equal volume of fresh solution. The OA concentration in samples was finally determined by HPLC-DAD. All studies were performed in triplicate.

To evaluate the kinetics and mechanism of drug release from the microemulsions, the Korsmeyer–Peppas model, Hixson–Crowell model and Higuchi model, and the first order and zero order mathematical models, were used, and the best-fitted model was selected based on high regression coefficient (R^2^) value for the release data.

#### 2.4.5. In Vitro Parallel Artificial Membrane Permeability Assay (PAMPA)

The test was carried out in a 96-well, MultiScreen-IP PAMPA (Millipore corporation) filter plate in order to evaluate the ability of compounds to diffuse from a donor compartment into an acceptor compartment. EtOH:PBS (30:70) mixture was selected as the acceptor medium for ME-1-OA and EtOH:PBS (5:95) for ME-2-OA. Each membrane was activated with a combination of lecithin and cholesterol, 10 g/L and 8 g/L, respectively, in 1,7-octadiene. Immediately after the deposition of the solution (5 µL), 250 µL of OA solution and appropriately diluted MEs-OA were added to each well of the donor plate. Each receptor plate was filled with 250 µL of acceptor medium. The plate was incubated at room temperature for 1 h. Then, the samples were withdrawn, properly diluted with methanol, centrifuged for 10 min at 14,000× *g*, and the OA concentration determined by HPLC-DAD. The permeability coefficient Pe (cm/s) was calculated according to the following equation:

$$P_{e}=-\frac{\ln\;\left[1-\frac{C_{At}}{C_{eq}}\right]}{A\;\left(\frac{1}{V_{D}}+\frac{1}{V_{A}}\right)t\;}$$

where A is the active surface area (0.3 cm^2^ × apparent porosity of the filter), V_D_ and V_A_ the well volume of the donor and acceptor plate (mL), respectively, t the incubation time (s), and C_At_ and C_Dt_ the concentration of OA in the acceptor and donor plate at time t, respectively. C_eq_ was calculated according to:

$$C_{eq}=\frac{C_{Dt}\;\times \;V_{D}+C_{At\;}\times \;V_{A}}{V_{A}+V_{D}}$$

The experiments were performed in triplicates.

### 2.5. Cell-Based In Vitro Experiments

#### 2.5.1. Cell line and Culture Conditions

The murine macrophages RAW 264.7 cell line (ATCC TIB-71™) was used in this study. Cells were grown in DMEM culture medium supplemented with L-glutamine (2 mM), streptomycin (100 µg/mL) and penicillin (100 U/mL), and 10% Heat-Inactivated Fetal Bovine Serum (HI-FBS), at 37 °C in 5% CO_2_ atmosphere. HI-FBS was obtained by incubating FBS at 55 °C for 30 min. Upon reaching 90% confluence, macrophage subcultures were prepared by scraping cells and adding appropriate dilutions of the cell suspension to a new plate. The culture medium was replaced every 3 days.

In in vitro cell-based experiments, cells were incubated in serum-free DMEM medium (starvation medium) for 6 h and then incubated for an additional 18 h in the presence of LPS (1 μg/mL). LPS was used as a pro-inflammatory and pro-oxidant stimulus. Cells untreated and not exposed to LPS stimulus were used as controls [35].

#### 2.5.2. MTT Assay

The Methyl Thiazolyl Tetrazolium (MTT) assay was used to assess the viability of RAW 264.7 cells under various experimental conditions [35]. Cells were seeded in a 96-well plate (15 × 10^3^ cells/well) in complete medium and incubated overnight. Next, cells were starved for 6 h, and then treated for an additional 18 h with OA and OA loaded in the two different formulations (ME-1-OA and ME-2-OA) at various concentrations, in the absence or presence of LPS (1 μg/mL). Cells treated with the empty ME-1 and ME-2 carriers were used as controls. Subsequently, cells were incubated with a 0.5 mg/mL solution of MTT (100 μL/well) for 1 h in the dark at 37 °C. After a wash in PBS, the insoluble formazan crystals were dissolved by adding 100 μL/well of dimethyl sulfoxide (DMSO). The iMARK microplate reader (Bio-Rad, Hercules, CA, USA) was used to measure absorbance values at a wavelength of 595 nm. Data were reported in percentage terms compared with untreated control cells not exposed to LPS. The experiment was repeated in triplicate.

#### 2.5.3. Intracellular ROS Production Detection

DCFDA fluorescent probe was used for sensitive and rapid quantification of intracellular ROS in response to LPS stimulus [35]. Cells were seeded in a 96-well plate (20 × 10^3^ cells/well) in complete medium and incubated overnight. After 6 h starvation, cells were treated for an additional 18 h with OA and OA loaded in the two different formulations (ME-1-OA and ME-2-OA) at various concentrations, in the absence or presence of LPS (1 μg/mL). Cells treated with the empty ME-1 and ME-2 carriers were used as controls. Next, DCFDA probe (10 μM in PBS) was added to each well and incubated in the dark for 1.5 h at 37 °C. Fluorescence values were measured at excitation and emission wavelengths of 485 and 538 nm, respectively, using a fluorescence microplate reader (Fluoroskan Ascent^TM^ FL Microplate Fluorometer, Thermo Fisher Scientific, (Waltham, MA, USA). The level of intracellular ROS was normalized to cell viability. Data were reported in percentage terms compared with untreated control cells not exposed to LPS. The experiment was repeated in triplicate.

### 2.6. Statistical Analysis

One-way analysis of variance (ANOVA) followed by Tukey’s HSD post hoc test was used to analyze the in vitro data. The threshold for statistical significance was set at *p* < 0.05.

## 3. Results

### 3.1. Preparation of Microemulsions

#### 3.1.1. Solubility Studies

A solubility test was conducted to select appropriate components for the preparation of MEs with a high drug-loading capacity. Different oils and surfactants were screened.

An increase in terms of OA solubility is evident for all considered oils, in particular with Capmul PG-8/NF, Capmul PG-12/NF, Capryol 90, Labrasol, Labrasol ALF, Laurogycole 90, Nigella Oil, and Isopropyl myristate (Table 1). Less promising results were observed in the case of Ethyloleate, Soybean Oil, and Triacetin. Surfactant selection is critical for the successful formulation of MEs, as it contributes to the reduction of interfacial tension by forming a film at the oil–water interface. In the present study, OA displayed a higher solubility in Transcutol HP and Cremophor EL, and to a lesser extent for PEG 400, Tween 20 and Tween 80.

Based on the results, for the development of ME-1, Capmul PG-8/NF was selected as the oily phase and Tween 20 as the surfactant. For the development of ME-2, Isopropylmyristate and Nigella sativa oil were selected as the oily phase. Cremophor EL was selected for use in further studies due to its solubility profile and its low toxicity level as a non-ionic surfactant. Transcutol HP was used in both the MEs.

#### 3.1.2. Pseudoternary Phase Diagrams

**Figure 1:**
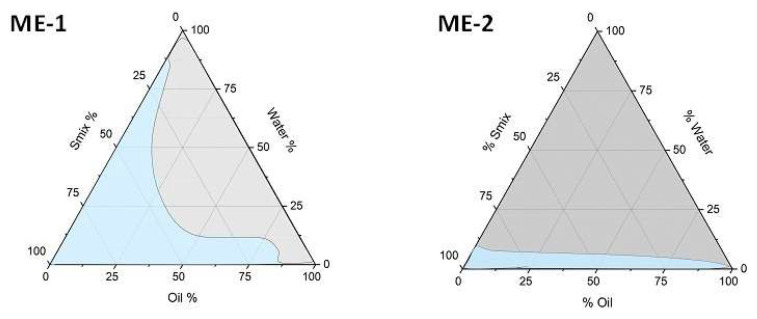
Pseudoternary phase diagrams of ME-1-OA and ME-2-OA.

Psuedoternary phase diagrams were constructed to select the field of existence of the Mes. Tween 20 and Transcutol HP for ME-1, and Cremophor EL and Transcutol HP in the case of ME-2, were mixed at different ratios under magnetic stirring to obtain the surfactant mixtures (Smix). After that, the pseudo-ternary phase diagrams were constructed by using water titration method with different combinations of oily phase and Smix. Figure 1 shows the pseudoternary phase diagrams of ME-1 and ME-2.

The ME domain (light blue) was determined by visual inspection. The rest of the region (grey) represents an emulsion (Figure 1). The final composition of the two MEs selected for OA loading is reported in Table 2.

After the elaboration of the pseudoternary phase diagrams, the maximum loading content was evaluated by adding an increasing amount of OA to the MEs under stirring at 50 ± 2 °C. ME-1 formulation was able to load up to 1 mg/mL of OA, and ME-2 incorporated 3 mg/mL of OA, without phase separation or precipitation phenomena.

### 3.2. Characterization of Microemulsions

#### 3.2.1. Particle Size and ζ-Potential Measurements

Microemulsion systems were prepared by mixing oil with surfactants, and water was added dropwise into oily phases under magnetic stirring. Empty and OA-loaded formulations were physically characterized by dynamic light scattering (DLS) and electrophoretic light scattering (ELS). The analyses confirmed the presence of a homogeneous system with narrow size distribution and appropriate values of polydispersity index (PdI). The presence of the OA did not affect the physical characteristics of the systems. MEs showed very small particle size (<100 nm) as shown in Table 3. Therefore, developed MEs are a successful tool to incorporate OA and to significantly ameliorate its solubility by 1000- and 3000-fold for ME-1 and ME-2, respectively, without destabilization of the system.

#### 3.2.2. TEM Analysis

**Figure 2:**
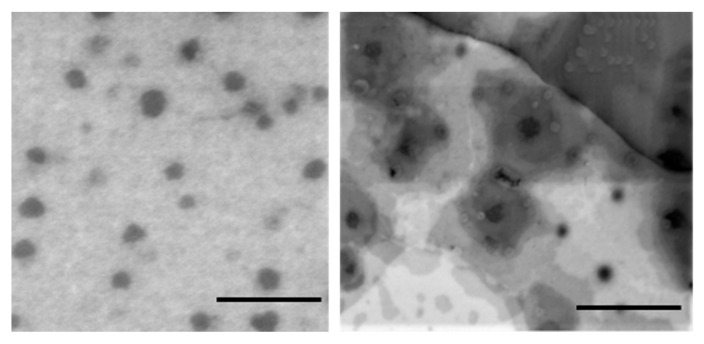
ME-1-OA (**left**) and ME-2-OA (**right**) TEM analysis. Bar: 500 nm.

TEM analysis (Figure 2) revealed separate single MEs’ droplets with a spherical shape, and with sizes in accordance with DLS data.

#### 3.2.3. Chemical and Physical Stability during Storage

**Figure 3:**
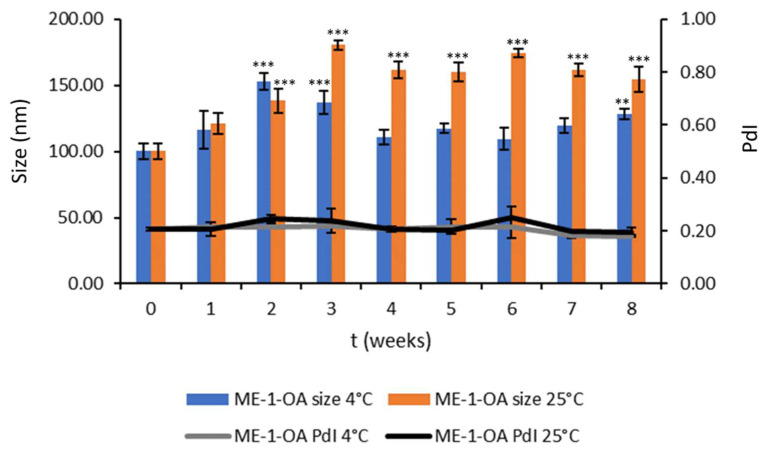
ME-1-OA storage stability study at 4 °C and 25 °C. Values are reported as mean ± SD of three independent experiments. Tukey’s test (*n* = 3): ** *p* < 0.01, *** *p* < 0.001 vs. time zero.

The OA-loaded MEs were stored in sealed glass containers at 4 °C and 25 °C for two months to evaluate the storage stability of the formulations. Periodically, chemical and physical stability were checked by visual inspection and the evaluation of the particle size, homogeneity, ζ-potential, and OA concentration. The formulation proved to be stable: no phase separation or creaming were observed. The size and homogeneity of ME-1-OA were mostly comparable after 8 weeks at 4 °C storage. Indeed, at 8 weeks the size of ME-1-OA was 129.47 ± 3.81 nm and the PdI of 0.18 ± 0.03. At 25 °C, ME-1-OA maintained the PdI unchanged, while starting from the second week there were significant changes in size, which still remained comparable. In fact, at 8 weeks, the size of ME-1-OA was 154.45 ± 9.69 nm and the PdI 0.19 ± 0.02 (Figure 3).

**Figure 4:**
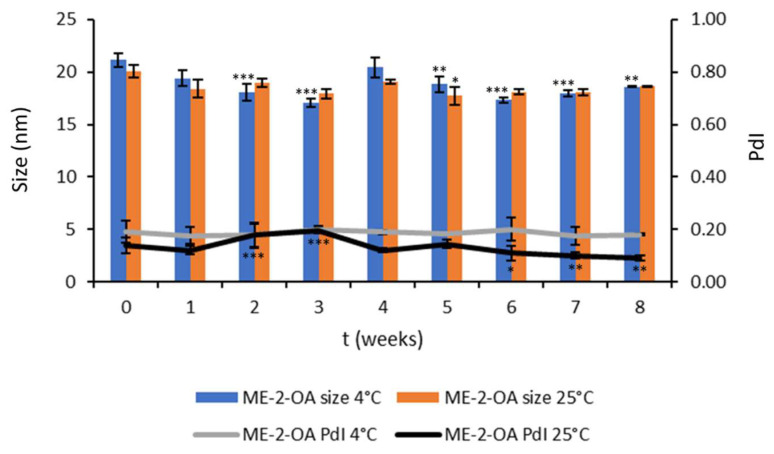
ME-2-OA storage stability study at 4 °C and 25 °C. Values are reported as mean ± SD of three independent experiments. Tukey’s test (*n* = 3): * *p* < 0.05, ** *p* < 0.01, *** *p* < 0.001 vs. time zero.

As for ME-2-OA, the chemical and physical stability during 8 weeks’ storage are reported in Figure 4. Notably, at 4 °C, the PdI of ME-2-OA remains unchanged over the 8 weeks, while significant changes in the size of ME-2-OA were observed from the second week, which, however, remained comparable with each other over time. In fact, at 8 weeks ME-2-OA showed a size of 18.61 ± 0.3 nm and PdI 0.18 ± 0.04. Even at 25 °C, the ME-2-OA remained stable over the 8 weeks, with retention of size (18.71 ± 0.2343 nm) and only small changes in PdI (0.0932 ± 0.010) (Figure 4).

The chemical stability of the developed formulations was confirmed through determination of the recovery percentage (R%) of OA compounds. Specifically, R% was 97.82± 3.37% for ME-1-OA and 101.8 ± 1.5% for ME-2-OA.

#### 3.2.4. In Vitro Release Studies

**Figure 5:**
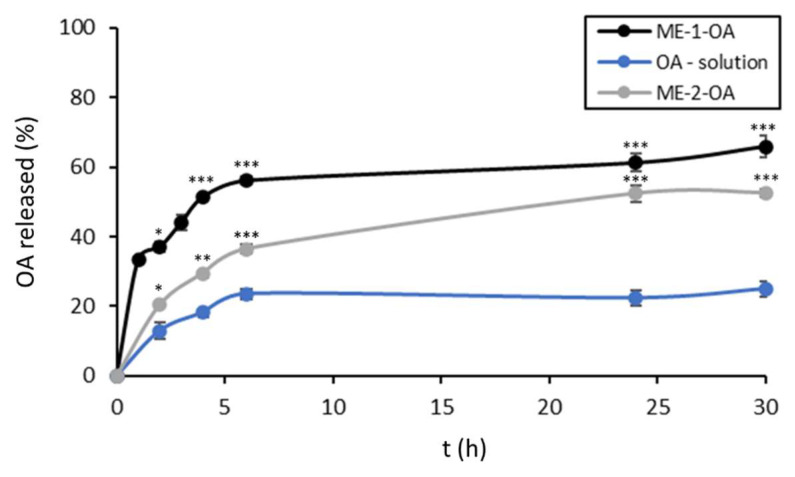
In vitro release profiles of OA from solution, ME-1-OA and ME-2-OA in EtOH:PBS (30:70). Values are reported as mean ± SD of three independent experiments. Tukey’s test (*n* = 3): * *p* < 0.05, ** *p* < 0.01, *** *p* < 0.001 vs. OA-solution at the corresponding times.

Comparing the release of formulated OA with free molecules, a greater release was achieved with both formulations. Free OA was gradually released from the solution over the first 6 h up to 20% (23.6 ± 1.25) and the plateau was maintained until 30 h (Figure 5). In the case of ME-1-OA, a fast release occurred during the first 4 h; indeed, the amount of OA released by ME-1-OA at 4 h was 51.40 ± 0.58%. Thereafter, OA release from ME-1-OA became more gradual and reached a plateau at 30 h, with a percentage of 65.87 ± 3.09%. OA release from ME-2-OA was slower than from ME-1-OA; in fact, at 6 h the percentage of OA released was 36.49 ± 1.45, and at 30 h it was 52.58 ± 2.26% (Figure 5).

An in vitro release study was also carried out in the SGF at pH 1.2 for 2 h, followed by SIF medium at pH 6.8 for 6 h (Figure S1 of Supplementary material) in order to simulate gastro-intestinal transit. The release profiles were similar to those in EtOH:PBS (30:70). In this case, the OA quantity of released was gradual and prolonged, and after 8 h, the percentage reached 60% and 51% for ME-1-OA and ME-2-OA, respectively. Additionally, in these media, the OA release from ME-2-OA was slower than ME-1-OA, probably due to the different components of the formulations.

Different theoretical models were considered to examine the nature of release in EtOH:PBS (30:70). The mechanism of drug release was defined by fitting the OA release data to various kinetics models (Table 4). Comparing the values of regression coefficient, the Higuchi model was the best to describe the kinetics of these two types of MEs.

#### 3.2.5. PAMPA Assay

Parallel artificial membrane permeability assay (PAMPA) is a fast model to predict in vitro passive transport permeability across the intestinal epithelium. Drug absorption through the gastrointestinal (GI) tract administered per *os* is one of the key factors in their bioavailability. Many properties regulate passive absorption through the GI tract, among them: log P, log D, molecular weight, ionization, and ability to form hydrogen bonds. All these parameters are useful in predicting passive uptake processes, but the use of in vitro artificial membrane assays proves useful in completing the prediction of passive uptake processes [36,37,38,39]. This non-cell-based model is widely contemplated as both robust and reproducible. In the present work, the ability of compounds to diffuse from a donor compartment into an acceptor compartment was evaluated. The permeability coefficient (Pe) of OA in solution was 3.69 ± 1.39 × 10^−7^ cm/s. The permeability of ME-1-OA and ME-2-OA was different than OA in solution; specifically, Pe of ME-1-OA was 5.70 ± 0.01 × 10^−6^ cm/s, while Pe of ME-2-OA was 4.74 ± 0.04 × 10^−5^ cm/s (*p* < 0.001). This resulted in a recovery of 98% for ME-1-OA and 94% for ME-2-OA.

The formulation improved the passive permeation of OA across the simulated membrane due to the increased solubility of the OA and the effect of penetration enhancers of the constituents of MEs, in particular Transcutol HP and Chremophor EL in ME-2-OA [40,41].

### 3.3. Effect of OA on Macrophages Cell Viability

**Figure 6:**
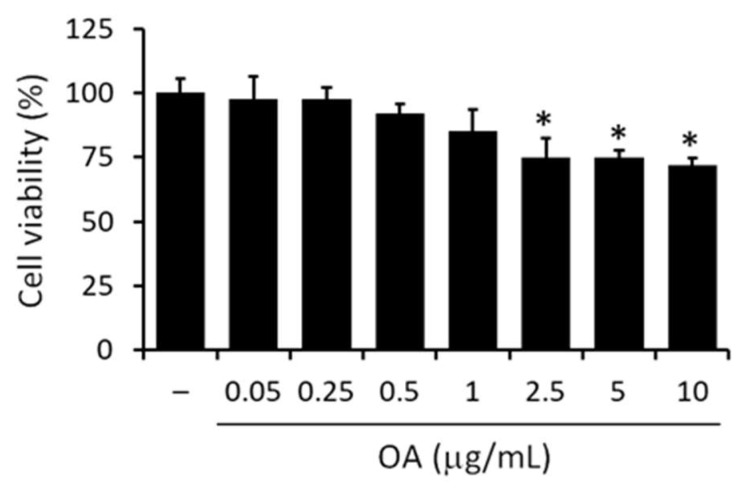
Effect of OA on RAW 264.7 cell viability. Cells were starved for 6 h and then treated for a further 18 h with doses of OA between 0.05–10 μg/mL. Values are expressed as percentages compared with untreated control cells (−). Data are reported as mean ± SD of three independent experiments. Tukey’s test (*n* = 3). * *p* < 0.05 vs. untreated control cells.

The MTT assay was used to determine the cytotoxicity of OA in RAW 264.7 macrophage cells over the wide range of concentrations between 0.05–10 μg/mL. As depicted in Figure 6, OA had a dose-dependent effect on cell viability, causing a 25–30% reduction in cell viability at concentrations between 2.5–10 μg/mL compared to untreated control cells. No significant change in cell viability was detected at lower doses of OA (0.05–1 μg/mL) compared to untreated control cells. Subsequent experiments were then performed at the nontoxic doses of OA (0.05–1 μg/mL).

### 3.4. Effect of OA on LPS-Induced Harmful Effects on Macrophages

**Figure 7:**
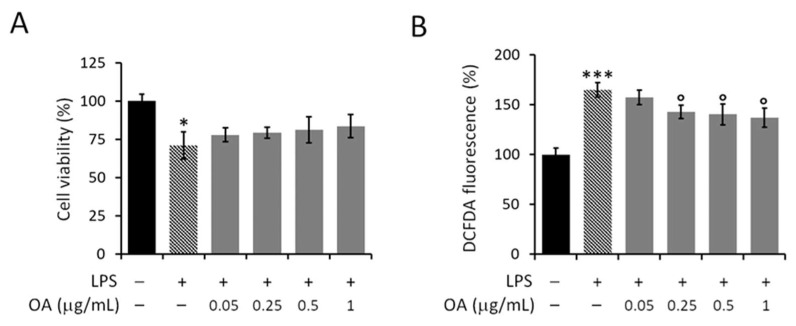
Effect of OA on LPS-induced harmful effects in RAW 264.7 cells, in terms of (**A**) cell viability and (**B**) intracellular ROS production. Cells were starved for 6 h and then treated for a further 18 h with nontoxic doses of OA (0.05–1 μg/mL) in the presence of LPS (1 μg/mL). Values are expressed as percentages compared with untreated (−) and LPS-unstimulated (−) control cells. Data are reported as mean ± SD of three independent experiments. Tukey’s test (*n* = 3). *** *p* < 0.001; * *p* < 0.05 vs. untreated (−) and LPS unstimulated (−) cells. * *p* < 0.05; *** *p* < 0.001 vs. untreated and LPS-unstimulated control cells. ° *p* < 0.05 vs. untreated but LPS-stimulated control cells.

LPS is a widely used stressogenic agent to stimulate and activate macrophages; evidence confirms that LPS triggers an innate immune response in which ROS-dependent oxidative stress is a critical mechanism [42]. As shown in Figure 7A, LPS caused a reduction in cell viability of approximately 30% (70 ± 7%) compared with unstimulated control cells. In addition, LPS determined a significant increase of about 65% (165 ± 7%) in intracellular ROS levels (Figure 7B). These data agree with the literature that describes free radical production as one of the mechanisms underlying the damaging effects of LPS [35,43,44]. This experimental model, widely reported in the literature, was used here to verify the potential protective effect of OA on the LPS-induced damaging effects. Cells stimulated with LPS were simultaneously treated with nontoxic doses of OA (0.05–1 μg/mL). However, as depicted in Figure 7A, OA had no significant protective effect against damaging effects from LPS. The levels of cell viability in LPS-stimulated and OA-treated cells were comparable to those of untreated but LPS-stimulated cells (Figure 7A). In contrast, the effect of OA observed at 0.25–1 μg/mL doses against LPS-induced intracellular ROS production was mild but significant (Figure 7B).

### 3.5. Bio-Enhancement of OA Activity Once Loaded into Microemulsions

Two different formulations of OA, ME-1-OA and ME-2-OA, were tested for potential bio-enhancement in OA activity against LPS-induced damaging effects on macrophages. The two MEs were loaded with 1 mg/mL and 3 mg/mL of OA in ME-1 and ME-2, respectively. The goal was to test the effect of ME-1-OA and ME-2-OA against LPS-induced damaging effects at appropriate dilutions, i.e., using a final concentration of loaded OA between 0.05–1 μg/mL (corresponding to the nontoxic doses of free OA).

However, to exclude any biological effect of the formulations at these dilutions, the effect of empty ME-1 and ME-2 was evaluated on RAW 264.7 macrophage cell viability. Specifically, ME-1 was shown to inhibit macrophage viability at higher doses (corresponding to ME-1-OA 0.5 and 1 μg/mL) and to be nontoxic at lower doses (corresponding to ME-1-OA 0.05 and 0.25 μg/mL), whereas ME-2 did not affect cell viability within all tested dilutions (Figure S2 of Supplementary Materials). Excluding toxic doses of ME-1, the effect of ME-1 (corresponding to ME-1-OA 0.05 and 0.25 μg/mL) and ME-2 (corresponding to ME-2-OA 0.05–1 μg/mL) on macrophage cell viability and ROS production in the presence of LPS was evaluated. None of the tested doses of empty carriers were able to counteract the LPS-induced damaging effects (Figure S3 of Supplementary Materials).

Thus, the potential bio-enhancement of OA activity was evaluated at loaded OA concentrations of 0.05 and 0.25 μg/mL for ME-1-OA, and 0.05–1 μg/mL for ME-2-OA.

**Figure 8:**
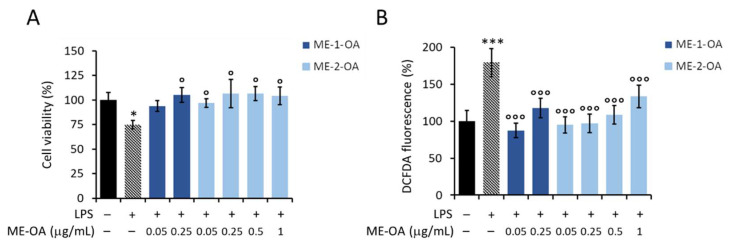
Effect of OA loaded into ME-1 and ME-2, i.e., ME-1-OA and ME-2-OA, respectively, on LPS-damaging effects in RAW 264.7 cells. (**A**) MTT assay and (**B**) detection of intracellular ROS production in cells starved for 6 h and then treated for 18 h with ME-1-OA at 0.05 and 0.25 μg/mL OA loaded (blue bars), and with ME-2-OA at 0.05–1 μg/mL OA loaded (light-blue bars), in the presence of LPS (+). Values are expressed as percentages compared to untreated (−) and LPS-unstimulated (−) control cells. Data are reported as mean ± SD of three independent experiments. Tukey’s test (*n* = 3). * *p* < 0.05; *** *p* < 0.001; vs. untreated and LPS-unstimulated control cells. ° *p* < 0.05; °°° *p* < 0.001 vs. untreated but LPS-stimulated control cells.

As shown in Figure 8A, encapsulated OA was able to significantly protect RAW 264.7 macrophage cells from LPS-induced toxicity, maintaining cell viability at levels comparable to untreated and unstimulated control cells. Despite the apparent protection, ME-1-OA at 0.05 μg/mL dose was not significantly protective against LPS cellular toxicity (Figure 8A, blue bar). However, OA carried by both ME-1 and ME-2 was able to prevent the LPS-induced increase in intracellular ROS levels, maintaining ROS at basal levels as untreated and unstimulated control cells (Figure 8B).

Taken together, these results suggest that ME-OA results in a bio-enhancement of the antioxidant protective action of OA against the LPS-induced damaging effects on RAW264.7 macrophage cells.

## 4. Discussion

A huge body of literature suggests that ROS-mediated oxidative stress and inflammation are two closely related pathophysiological phenomena and the lowest common denominator of most chronic and noncommunicable diseases [1,2]. Therefore, the importance of blocking excessive ROS production triggered by stressors is increasingly recognized. Antioxidant strategies could contribute to the prevention of chronic diseases. There is increasing evidence that phytochemicals possess bioactive properties that can maintain the balance between ROS production and their scavenging [45]. OA, a phytochemical belonging to the triterpenoid class, is known for its innumerable biological properties that make it a good candidate for potential alternative and complementary therapies for the treatment and management of several diseases [22]. Among its multiple mechanisms of action, OA stands out for its antioxidant property by acting not only as a free radical scavenger through direct chemical reactions but also as a biological molecule by quenching ROS, inhibiting lipid peroxidation or stimulating cellular antioxidant defenses [46]. Despite this, the clinical applications of OA are still quite limited due to issues with water solubility, stability and bioavailability.

MEs are a drug delivery system composed of oil, surfactant, cosurfactant and aqueous phases, and they are good candidates for oral administration due to their ability to improve the solubility and the absorption of lipophilic compounds [32,47]. The enhancing absorption in the gastrointestinal tract is caused by surfactants that act as permeation enhancer and modulate the fluidity of the mucosal membrane [48].

The selection of excipients is important to obtain the desired formulation, with a good stability and loading capacity. Lipid excipients are widely used and their characteristics influence the absorption process [49]. Different factors affect the choice of excipients for lipid-based formulations, such as purity and chemical stability, miscibility, and solubilization properties. When developing oral MEs, special attention must be paid to the choice of components; in fact, it is essential to increase the oral bioavailability of the drug while maintaining safety of use. Excipients should be chosen from the list of excipients generally recognized as safe. The economic cost of nanomaterials is another critical factor for their application in therapy. Selected excipients have an established use in pharmaceuticals and food, are not expensive, and some are already used in commercial products.

In our study, we selected food-grade ingredients, and based on solubility studies, Capmul PG-8/NF was used for ME-1 formulation as the oily phase and Tween 20 and Transcutol HP as surfactants. Capmul PG-8/NF is the propylene glycol monoester of caprylic acid. It has already been used as an oily phase in other studies for its ease of emulsification and has been widely used for the development and optimization of nanoemulsions/microemulsions/self-nanoemulsifying drug delivery systems (SNEDDS)/self-microemulsifying drug delivery systems (SMEDDS) of various poorly soluble drugs. Tween 20 is a non-ionic surfactant widely used in the field of MEs with high stability, low toxicity, low irritation, and possible biodegradability [50]. It is generally considered safe and approved for use in many drugs, cosmetics, and foods because it is nonirritant and has low toxicity [51].

Transcutol HP is a hydrophilic surfactant and increases ME formation, promoting emulsification of surfactants, and has a great solubilizing capacity. It is widely used in oral, transdermal, and topical formulations as a solubilizer and absorption enhancer [52].

Isopropyl myristate and *Nigella sativa* oil were selected as the oily phase, and Cremophor EL and Transcutol P as surfactants, for the preparation of ME-2. Nigella oil is obtained by cold-pressing the seeds of *Nigella sativa* or black cumin. It is rich in fatty acids such as palmitic acid, oleic acid and linoleic acid [53]. Isopropyl myristate is the ester of isopropyl alcohol and myristic acid. It has usually been used for topical pharmaceutical preparations, but oral application in the preparation of SMEDDS or microemulsions has also been reported [54,55,56].

Cremophor EL is a complex mixture of both hydrophobic and hydrophilic components, with the main components being glycerol polyethylene, glycol ricinoleate and glycerol ethoxylates, respectively. It was selected due to its solubility profile and its low toxicity level as a non-ionic surfactant.

In this study, the two MEs had a good solubilizing effect on OA without destabilization of the system. The most marked effect on solubility and permeability observed with ME-2 is due to the improved ability of Cremophor to act as a solubilizer.

The optimized formulations were suitable in terms of physico-chemical characteristics for oral administration. Isopropyl myristate and Nigella oil as an oily phase, and Cremophor as a surfactant, determined the lower size of ME-2-OA in respect to ME-1.OA. The droplet size of the microemulsion is greatly affected by the oily phase, type of surfactant and cosurfactant and their ratio [57,58]. The addition of surfactants to the microemulsion systems causes the interfacial film to stabilize and condense, while the addition of cosurfactant causes the film to expand [58]. The cosurfactant could form more stable interfacial film with the surfactants, which will further lower the interfacial tension between the oil and water phases, fluidize the hydrocarbon region of the interfacial film, and modify the film curvature [33]. The different droplet size was also attributed to the complex structure and affinity of the drug to the components of the nanoemulsion. Cremophor was also reported to significantly decrease the droplet size [58]. The size was also affected by the nature and the concentration of the oil. An increase in the percentage of the oil induced the increase of the mean droplet size [58,59]. Some of the co-surfactants are also known as penetration enhancers. Tensides act through fluidizing the membranes of living cells [60]. These properties, together with the small size, can explain the improved in vitro permeability of formulated OA with both artificial membranes and RAW 264.7 murine macrophage cells.

Both MEs showed appropriate stability for more than 60 days during storage at 4 °C and 25 °C. ME-2-OA was the most stable formulation, showing constant physical and chemical parameters after 8 weeks at both temperatures. ME-1-OA was slightly less stable than ME-2-OA. It was more stable at 4 °C, with a small increase in size, but with constant PdI and recovery values. Both systems had a pronounced effect in terms of in vitro release compared to free OA. OA release from the formulations was significantly higher and faster than from the solution. This is an important factor in increasing the absorption and bioavailability of OA in the aqueous phase. Furthermore, the OA release from ME-2-OA was slower than ME-1-OA, both in EtOH:PBS 30:70 and in simulated gastro-intestinal fluids. This is probably related to different components of the formulations. Similar percentages and release profiles have been reported in the literature for nanoemulsions, SNEDDS and micelles optimized for OA delivery [58,59,61,62,63].

Comparing the values of regression coefficient values in EtOH:PBS 30:70, the Higuchi model was the best to describe the kinetics of these two types of MEs, suggesting that the drug release occurs by diffusion [64,65].

MEs also improved the passive permeation of OA as evidenced by PAMPA experiments, in particular in the case of ME-2-OA. Cremophor and Transcutol are known for their properties as solubilizing agents, absorption enhancers and P-gp inhibitors [66,67]. Therefore, ME-2-OA provided enhanced permeability of OA in the PAMPA test and ameliorated activity in the cellular test. In the PAMPA, Pe of ME-2-OA was an order of magnitude greater than ME-1-OA. The permeability increases due to the increased solubility of the OA and to the presence of surfactants used as stabilizers of the internal phase. Furthermore, as previously reported, the surfactants enhance drug permeability in many ways, such as by increasing transcellular permeability and by inhibiting the efflux transport systems [32,68]. Moreover, non-ionic surfactants also contributed to increasing the contact time with the absorption site, and to increase endocytic and transcellular pathways by opening the tight junctions [69]. It was indicated that Cremophor and Transcutol may inhibit the function of P-gp by affecting membrane fluidity [70]. Furthermore, the small dimensions of droplets of ME-2-OA characterized by a big surface area also increase the solubility and absorption of the drug. These aspects increase the penetration into the cells and could also positively influence the bioavailability of the OA.

Here, RAW 264.7 murine macrophages were used as an experimental cell model to evaluate the effect of OA and formulated OA against LPS-induced cytotoxicity and ROS production. LPS was used to activate macrophages by triggering a sharp increase in intracellular ROS levels, and thus generating an oxidative stress condition. Initially, in vitro detection techniques were used to evaluate the effect of free OA against the LPS-induced cellular response. The obtained results revealed that free OA, at non-toxic doses, had only a very mild inhibitory effect on LPS-induced ROS production, and no protective effect against the LPS-induced cytotoxicity.

A possible bio-enhancement of OA activity against the damaging LPS stimulus was therefore evaluated using the two different OA-loaded MEs. Excluding the toxic doses of the empty carriers, in this work it was obtained that, indeed, carrier-loaded OA had a significant protective effect against both LPS-induced cytotoxicity and intracellular ROS formation.

Recently, it has been described that a ME in some cell lines can promote the penetration of substances with poor solubility and bioavailability, which is due to surfactants that increase permeability across the cell membrane [71]. Therefore, we could speculate that OA bio-enhancement, here observed, may be due to enhanced internalization of OA by the microemulsion system. The surfactants decrease the interfacial tension of oil/water to a very low value, which facilitates the dispersion process in the preparation of ME and the formation of a flexible film around the droplets. The small droplet sizes containing OA promote wide dispersion of the drug and also increase the release into the water phase faster than ordinary OA.

In particular, we can propose ME-2-OA as a better vehicle for OA delivery because of both its small size and composition. In fact, the small size of ME-2-OA compared with ME-1-OA could influence fusion kinetics by favoring internalization by endocytosis. In fact, smaller size formulations have a better rate of fusion with the cell membrane than larger size formulations [71].

Therefore, this work suggests microemulsions, particularly ME-2, as a viable delivery system for OA to overcome its limitations in pharmaceutical applications.

## 5. Conclusions

Despite the innumerable biological properties of OA, its potential use in pharmaceuticals is limited by its poor solubility in water and consequent low bioavailability.

In this study, OA was effectively incorporated into two different microemulsions, i.e., dispersed systems consisting of two immiscible phases that promote rapid solubilization and absorption in the gastrointestinal tract.

The proper physico-chemical characteristics, improved OA solubility, and its passive permeability through an artificial membrane were demonstrated with the newly formulated systems. Specifically, ME-2-OA increased the solubility of OA by 3000-fold, while greater OA release was observed for both MEs compared with the control solution at different pH.

In addition, preliminary in vitro experiments on murine macrophages, discussed here, showed that MEs enhanced the protective antioxidant effect of OA against LPS-induced stress in macrophages. Since OA, a plant triterpenoid, is present in many foods and used in traditional medicine for a variety of diseases, MEs could be an interesting and practical formulation to enhance the oral bioavailability of OA and thus evaluate its potential clinical interest for the treatment of some chronic and noncommunicable diseases.

## Figures and Tables

**Table 1:** Saturation solubility (mg/mL) of oleanolic acid in different oils and surfactants.

Solvent	Solubility	Solvent	Solubility
Capmul PG-8/NF	19.72 ± 0.13	Isopropil myristate	9.44 ± 1.05
Capmul PG-12/NF	16.42 ± 1.04	Lauroglycole 90	10.75 ± 0.56
Captex 300	3.74 ± 0.10	Nigella oil	35.40 ± 1.41
Captex 355	3.19 ± 0.24	Sunflower oil	11.75 ± 0.46
Capryol 90	24.95 ± 0.90	Soybean oil	1.06 ± 0.17
Cremophor EL	28.32 ± 0.93	PEG 400	8.49 ± 0.58
Labrafac Lipophile	13.29 ± 0.87	Transcutol HP	41.02 ± 2.41
Labrasol	13.27 ± 0.21	Triacetine	3.09 ± 0.17
Labrasol ALF	11.98 ± 0.84	Tween 20	6.19 ± 0.58
Etyloleate	2.13 ± 0.21	Tween 80	4.56 ± 0.36

**Table 2:** Compositions (*w/w*%) of ME formulations.

Formulation	Capmul	Isopropyl Myristate/ Nigella Oil (1:1)	Tween 20	Transcutol HP	Cremophor EL	Water
ME-1	6		17	37		40
ME-2		4		30	16	50

**Table 3:** Characteristics of empty and OA-loaded ME formulations.

Sample	Size (nm) ± ds	PdI ± ds	ζ-Pot ± ds
ME-1	94.51 ± 2.17	0.21 ± 0.03	−4.15 ± 0.05
ME-2	15.62 ± 0.19	0.20 ± 0.07	−9.87 ± 0.09
ME-1-OA	93.01 ± 3.37	0.20 ± 0.04	−3.32 ± 0.02
ME-2-OA	17.62 ± 0.23	0.20 ± 0.07	−11.63 ± 0.01

**Table 4:** Regression coefficient (R^2^) obtained in different kinetics models for OA release from ME-1-OA and ME-2-OA.

Release Kinetics	ME-1-OA	ME-2-OA
Zero order	0.4812	0.7518
First order	0.6326	0.8402
Korsmeyer–Peppas	0.4527	0.6329
Hixson	0.5822	0.8129
Higuchi	0.7194	0.9320

## Data Availability

The data presented in this study are available on request from the corresponding author. Samples of the ME-1-OA and ME-2-OA are available from the authors.

## Supplementary Materials

The following supporting information can be downloaded at: https://www.mdpi.com/article/10.3390/pharmaceutics14102232/s1, Figure S1: In vitro release profile of OA from the ME-1-OA and ME-2-OA in SGF (pH 1.2, 2 h) and (SIF) (pH 6.8, 6 h). Each value is the mean ± SD of three separate determinations; Figure S2: Effect of empty carriers, ME-1 and ME-2, on RAW 264.7 cell viability. MTT assay on cells starved for 6 h and then treated for 18 h with empty carriers at appropriate dilutions. Forconvenience ME-1 or ME-2 at dilutions corresponding to 0.05, 0.25, 0.5, 1 µg/mL ME-1-OA or ME-2-OA are referred to here as (a), (b), (c), and (d), respectively. Values are expressed as percentages with respect to untreated control cells (−). Data are reported ad mean ± SD of three independent experiments. Tukey’s test (*n* = 3). *** *p* < 0.001 vs. untreated control cells.; Figure S3: Effect of empty carriers, ME-1 and ME-2, on LPS-damaging effects in RAW264.7 cells. Cells were starved for 6 h and then treated for 18 h with empty carriers at appropriate dilutions in the presence of LPS (+). For convenience ME-1 or ME-2 at dilutions corresponding to 0.05, 0.25, 0.5, 1 µg/mL ME-1-OA (blue bars) or ME-2-OA (light-blue bars) are referred to here as (a), (b), (c), and (d), respectively. Values are expressed as percentages with respect to untreated (−) and LPS-unstimulated (−) control cells. Data are reported as mean ± SD of three independent experiments. Tukey’s test (*n* = 3). *** *p* < 0.001 vs. untreated and LPS-unstimulated control cells.
