# Supplementary material for: Microemulsions Enhance the In Vitro Antioxidant Activity of Oleanolic Acid in RAW 264.7 Cells
Source: Pharmaceutics. 2022 Oct 19;14(10):2232. doi: 10.3390/pharmaceutics14102232 (PMC9610975; doi:10.3390/pharmaceutics14102232)
Supplement: Supplementary file 1 [file pharmaceutics-14-02232-s001.zip › pharmaceutics-1939095-supplementary.pdf]

Supplementary Materials

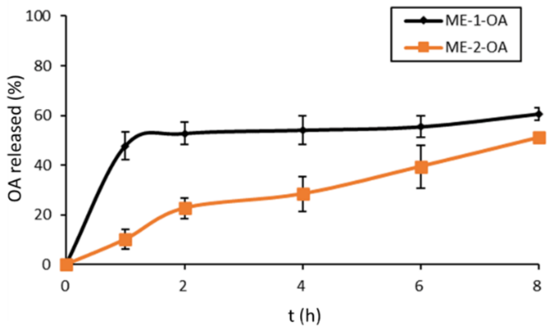

Figure S1. In vitro release profile of OA from the ME-1-OA and ME-2-OA in SGF (pH 1.2, 2h) and (SIF) (pH 6.8, 6 h). Each value is the mean ± SD of three separate determinations.

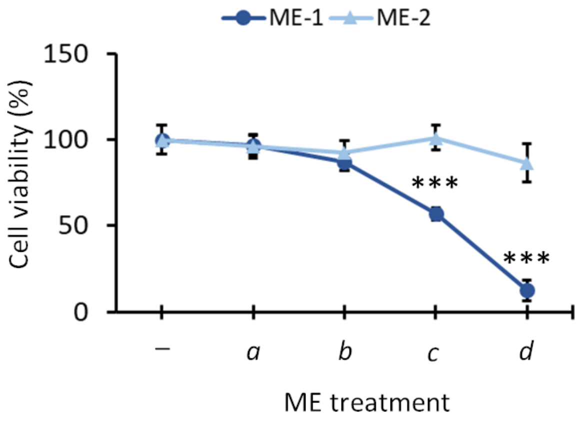

Figure S2. Effect of empty carriers, ME-1 and ME-2, on RAW 264.7 cell viability. MTT assay on cells starved for 6 h and then treated for 18 h with empty carriers at appropriate dilutions. For convenience ME-1 or ME-2 at dilutions corresponding to 0.05, 0.25, 0.5, 1 µg/mL ME-1-OA or ME-2-OA are referred to here as (*a*), (*b*), (*c*), and (*d*), respectively. Values are expressed as percentages with respect to untreated control cells (–). Data are reported ad mean ± SD of three independent experiments. Tukey's test (n=3). *** $p < 0.001$ vs. untreated control cells.

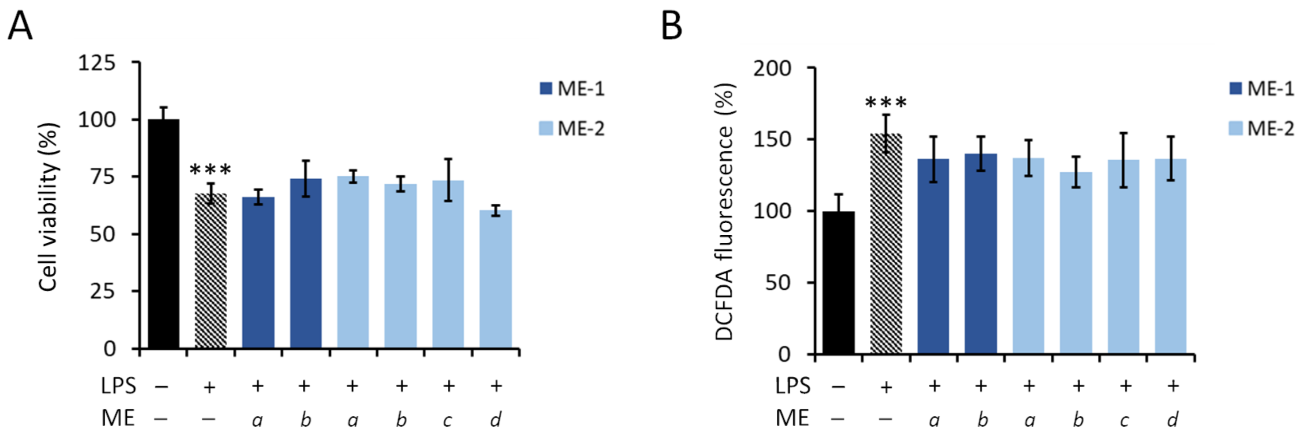

Figure S3. Effect of empty carriers, ME-1 and ME-2, on LPS-damaging effects in RAW264.7 cells. Cells were starved for 6 h and then treated for 18 h with empty carriers at appropriate dilutions in the presence of LPS (+). For convenience ME-1 or ME-2 at dilutions corresponding to 0.05, 0.25, 0.5, 1 µg/mL ME-1-OA (blue bars) or ME-2-OA (light-blue bars) are referred to here as (*a*), (*b*), (*c*), and (*d*), respectively. Values are expressed as percentages with respect to untreated (–) and LPS-unstimulated (–) control cells. Data are reported as mean ± SD of three independent experiments. Tukey's test (n=3). *** $p < 0.001$ vs. untreated and LPS-unstimulated control cells.
